# Microvascularized tumor organoids-on-chips: advancing preclinical drug screening with pathophysiological relevance

**DOI:** 10.1186/s40580-021-00261-y

**Published:** 2021-04-13

**Authors:** Jungeun Lim, Hanna Ching, Jeong-Kee Yoon, Noo Li Jeon, YongTae Kim

**Affiliations:** 1 School of Mechanical and Aerospace Engineering, Seoul National University, Seoul, 08826 Republic of Korea; 2 George W, Woodruff School of Mechanical Engineering, Georgia Institute of Technology, North Ave NW, Atlanta, GA 30332 USA; 3 Institute of Advanced Machinery and Design, Seoul National University, Seoul, 08826 Republic of Korea; 4 Institute of Bioengineering, Seoul National University, Seoul, 08826 Republic of Korea; 5 Parker H. Petit Institute for Bioengineering and Bioscience, Georgia Institute of Technology, Atlanta, GA 30332 USA; 6 Wallace H. Coulter Department of Biomedical Engineering, Georgia Institute of Technology, Atlanta, GA 30332 USA; 7 Institute for Electronics and Nanotechnology, Georgia Institute of Technology, Atlanta, GA 30332 USA

**Keywords:** Microfluidics, Tumor organoids, Microvasculature, Organ-on-a-chip

## Abstract

Recent developments of organoids engineering and organ-on-a-chip microfluidic technologies have enabled the recapitulation of the major functions and architectures of microscale human tissue, including tumor pathophysiology. Nevertheless, there remain challenges in recapitulating the complexity and heterogeneity of tumor microenvironment. The integration of these engineering technologies suggests a potential strategy to overcome the limitations in reconstituting the perfusable microvascular system of large-scale tumors conserving their key functional features. Here, we review the recent progress of in vitro tumor-on-a-chip microfluidic technologies, focusing on the reconstruction of microvascularized organoid models to suggest a better platform for personalized cancer medicine.

## Introduction

Cancer pathophysiology is extremely complex since the tumor microenvironment (TME) incorporates diverse factors, including extracellular matrix (ECM) and vascular constructs, with multiple stromal cell types [1]. Reproduction of the multiplex in vivo-like TME has been challenging due to the complexity and heterogeneity. The development of preclinical models pertinent to the pathophysiology of human cancers has contributed to clinical research for cancer therapeutics, and their roles are becoming more important [2]. Conventional preclinical animal models have been widely applied to anti-cancer drug development [3, 4]. However, they are restricted by a limited throughput, ethical issues, costly and time-consuming processes, and, more importantly, differences between animal and human physiology.

Recent advancement in 3D cell culture techniques to recapitulate human organ-specific microenvironment in vitro, which is known as organoids [5] or organs-on-a-chip [6], has shown great potential to overcome the limitations of conventional animal models. Organ-on-a-chip technologies and organoid models have emerged to better mimic the TME with human cells and to improve the efficiency with greater throughputs for more effective translational research for cancer treatment. TME-on-a-chip techniques have advantages for coculturing various cell types in TME under highly controlled dynamic microfluidic flow profiles to recapitulate essential physiological phenomena in TME, while organoid technologies enable the development and reconstitution of 3D tumors retaining the inherent traits and the function of intact tumor tissues and the patient-derived TME and address intra- and inter-patient heterogeneity. Nevertheless, these technologies remain to be further developed to better reconstruct the notorious perplexity of TME. For example, it remains elusive whether tumor cells construct vessel-like channels themselves and connect to normal blood vessels or growth factors secreted by tumor cells induce blood vessels to grow into the tumor tissue [7]. Elucidation of the mechanism of tumor vasculogenesis will contribute to modify the prognosis and therapy in cancer [8].

Recently, attempts to integrate organoids and organ-on-a-chip technologies have shown the potential to synergize with each other. Notably, microvasculature-on-a-chip techniques facilitate the establishment of vascular systems that have functional in vivo-like solid tumors created by organoid engineering methods. In this Review, we highlight recent developments of microvasculature-on-a-chip and tumor organoid formation and discuss how these techniques play an essential role in the research of vascular biology and oncology and in the development of cancer therapeutics. We review representative approaches using tumor organoids and microvasculature-on-a-chip to reconstruct a microvascularized tumor system. Finally, we discuss the future perspective and challenges of this integrative technology named “microvascularized tumor organoids-on-a-chip” in reflecting intrinsic cancer traits on the preclinical modeling to facilitate cancer research and therapeutics development.

## Tumor organoid

Over the past decades, advances in preclinical cancer models have contributed to the development of cancer research and therapeutics. Despite the remarkable progress in cancer treatment, many regimens have presented disappointing outcomes due to the incomplete elimination of cancer cells. This fact mainly stems from the lack of understanding of tumor heterogeneity, which incorporates intra- and inter-tumor differences in gene expression, proliferation, metastatic feature, morphology, phenotype, and mutational profiles [9]. To address the challenge, it is essential to translate knowledge of cancer development from bench to bedside.

Standard oncology models like animal models and two-dimensional in vitro culture systems have widely been used for innovative discoveries of pathogenesis and therapeutics in oncology in vivo [10–12]. They allow for profound exploration; however, most of them lack complex human cancer traits. Similarly, 2D in vitro cancer modeling does not truly reflect the pathological characteristics of the human TME. Patient-derived tumor xenografting (PDTX) models, which have been successful in replicating the significant portions of tumor multiplexity, compromise immune systems [13, 14].

Organoid technologies, which are 3D in vitro constructs developed from self-organizing stem cells or primary tissue emulating in vivo tissue, have advanced the development of more physiological human models for translational research, as well as developmental studies. Beyond the potential of organoids to mimic normal human tissue microenvironment, this technology has been employed in cancer research to facilitate understanding of oncology and cancer therapeutics, termed tumor organoid [15, 16]. To date, tremendous efforts using human cell sources have been made to develop preclinical tumor organoid models of human cancers, including gastrointestinal, prostate, brain, kidney, and breast cancers.

### Gastrointestinal cancer

Globally, about one-third of the entire incidence and mortality of cancer is associated with gastrointestinal cancers, which incorporates colorectal cancer, gastric cancer, liver cancer, and pancreatic cancer. So far, gastrointestinal tumor organoids have been cultured to recapitulate the functions and architectures of these cancer types.

#### Colorectal cancer

Worldwide, colorectal cancer, which is a tumor that develops from the colon or rectum, is one of the most common cancers [17]. Colorectal cancer modeling via tumor organoid has been developed using culture condition to enable the long-term culture of villus-like epithelial architectures [18] and CRISPR-Cas9-editing techniques [19–21] from human patient-derived tissue acquired by surgical resection or endoscopy biopsy, establishing an organoid library [22]; other colorectal tumor organoids derived from surgical resection were applied to high-throughput drug screening [23–25]. Furthermore, colorectal tumor organoids extracted by patient tissue biopsy of metastatic lesion were used to compare the reactions to anti-cancer agents with the responses to the patients in clinical trials [26] as well as stored in the collection [27, 28].

#### Gastric cancer

Gastric cancer remains the third most common cause of cancer-related deaths of all malignancies worldwide [29]. To emulate the pathophysiology of gastric cancer, a standard protocol for gastric tumor organoids formation was established to represent the traits of each of its four subtypes [30] and recapitulate most of the critical hallmarks of bacterial infection using surgically resected tissue of patients [31, 32]; CRISPR-Cas9-editing method was used to modify gastric cancer organoids to model each of the four subtypes of gastric cancer [33]. Using patient tissue specimen of the metastatic insult, a library of gastric tumor organoids to study chemosensitivity was demonstrated [26].

#### Liver cancer

Internationally, primary liver cancer is the fourth leading cause of cancer mortality [29]. The histological and genetic features and metastatic characteristics of hepatocellular carcinoma were replicated using surgically resected primary liver cancer patient tissue [34]. Besides, long-term hepatocellular carcinoma organoid cultures from tumor needle biopsies of liver cancer patients were established [35]. The organoids preserve the genetic heterogeneity, the tumor marker expression, and the morphology of the originating tumors presenting the application of the tumor organoids as a tool for testing cancer treatments.

#### Pancreatic cancer

The incidence of pancreas cancers continues to rise [17], one of the major causes of cancer death [36]. To establish pancreatic cancer modeling, surgically resected pancreatic cancer patient tissue derived from resected tumors or biopsies has been contributed to constructing pancreatic tumor organoids with retaining morphological heterogeneity and histological structure and building tumor organoids biobanks [37, 38]; therapeutic profiling was performed through the tumor organoids as a tool [39, 40]. Moreover, CRISPR-Cas9 technology enabled the reconstitution of progression from pancreatic intraepithelial neoplasia to adenocarcinoma using pancreatic cancer patient specimens [41, 42].

### Prostate cancer

Prostate cancer incidence and deaths have increased in the recent past few years [43, 44]. As prostate cancer modeling tools, the prostate tumor organoids were generated using metastatic human prostate cancer lesions and circulating tumor cells [45, 46]. The studies for prostate tumor organoids demonstrated that the organoids created from human patient tumor specimens with detailed characteristics were amenable to drug testing [45] and study of prostate cancer initiation [47].

### Brain cancer

Among adolescents and children, brain cancers, including glioma, glioblastoma, and medulloblastoma, are the most common cancer and one of the leading causes of cancer-specific death [48]. Brain tumor organoid models derived from human embryonic stem cells were built via tumorigenic mutations using transposon and CRISPR-Cas9 technologies to study the human brain tumor developmental process incorporating initiation and progression [49]. Resected tissues of glioblastoma patients were utilized to build glioblastoma organoids to reconstitute hypoxic gradients, and stem cell heterogeneity was constructed [50]. Interestingly, glioblastoma modeling was developed using human embryonic stem cells-derived cerebral organoids with invasion of patient-derived glioma stem cells, which replicated the biological behaviors, genetic features, and phenotypes of human glioblastoma [51].

### Kidney cancer

In the United States, at least 300,000 kidney cancer survivors have or will develop chronic kidney disease [52]. Renal carcinoma organoids recapitulating the phenotypic traits were generated using human tissue specimens [53, 54]. Also, the pediatric kidney cancer tissues extracted through nephrectomy or biopsy were utilized to derive kidney tumor organoids from retaining phenotypic and histological characteristics of the parental tumor tissue [55].

### Breast cancer

Among females, breast cancer is the leading cancer and the main cause of cancer-related death [29]. Human breast tumor biopsies were applied to establish breast tumor organoids to reconstruct the basement membrane [56] and identify drug response [57]. In addition, a living biobank of breast tumor organoids to maintain genetic and histological traits of parental tumors was built, which enabled high-throughput drug screening [58]. As one of the breast cancer types, the papillary carcinoma organoids to mimic the histological features and the biomarker expression were created using patient tumor tissues [59].

### Others

Various types of tumor organoids have been engineered to reconstruct specific microenvironments in tumors, such as the immune system. For instance, air-liquid interface tumor organoids derived from surgically resected patient tumor tissue emulated the programmed cell death protein 1/ programmed death-ligand 1 (PD-1/PD-L1)-dependent immune checkpoint, which is a key regulatory physiological immune checkpoint [60]. Furthermore, the platform for the implementation of personalized high-throughput drug screening with whole-exome sequencing analysis was constructed via patient-derived multiple types of cancer organoids [61].

## Microvasculature-on-a-chip

Microvascular networks are composed of terminal arterioles, capillaries, and postcapillary venules, where the blood flow and drainage occur. The microvascular system is essential in human physiology as a circulatory network to deliver nutrients and oxygen and eliminate the waste products and CO_2_ by constructing inter-organ connections [68]. Since this circulatory system plays an integral part in human metabolism and is immensely associated with pathophysiology, it is crucial to reconstitute the structure in preclinical models. Reconstructing the microvascular architecture and function, such as endothelial barriers and vascular perfusability, has been challenging for the development of conventional in vitro preclinical models.

To overcome the limitations, organ-on-a-chip systems provide for a controllable 3D tissue culture module, allowing to emulate the microvascular network. The 3D microfluidic in vitro model has enabled the culture of microvasculature in a dynamic microenvironment, termed microvasculature-on-a-chip. Recently, the dynamic stimulation, such as the blood flow-mimicking shear stress, advanced the physiological relevance in terms of its function, morphology, and junction expression. Here we reviewed microfluidic in vitro models to reconstruct the microvasculature using human endothelial cells (ECs) by categorizing them based on engineering aspects: self-assembled microvascular network, EC monolayer, and tubular endothelial barrier.

### Self-assembled microvascular network

A network of blood vessels establishes the circulatory system, regulating the systemic process by transporting vital substances. Through the circulatory path, drugs are distributed throughout the body. Of particular interest is the study to replicate the essential microvascular organization related to multiple pathologies and drug treatment. The adjusted microenvironments by engineering cellular elements, extracellular matrix, mechanical stress, and other factors enable the reconstitution of the connected microvascular network with a lumenized structure via the self-organizing ability of ECs. This type of in vitro model allows for the construction of blood vessels having diameters in tens of micrometers. Also, this platform emulates fundamental mechanisms in microvasculature formation, known as angiogenesis and vasculogenesis, in the orchestrated microenvironment.

**Fig. 1:**
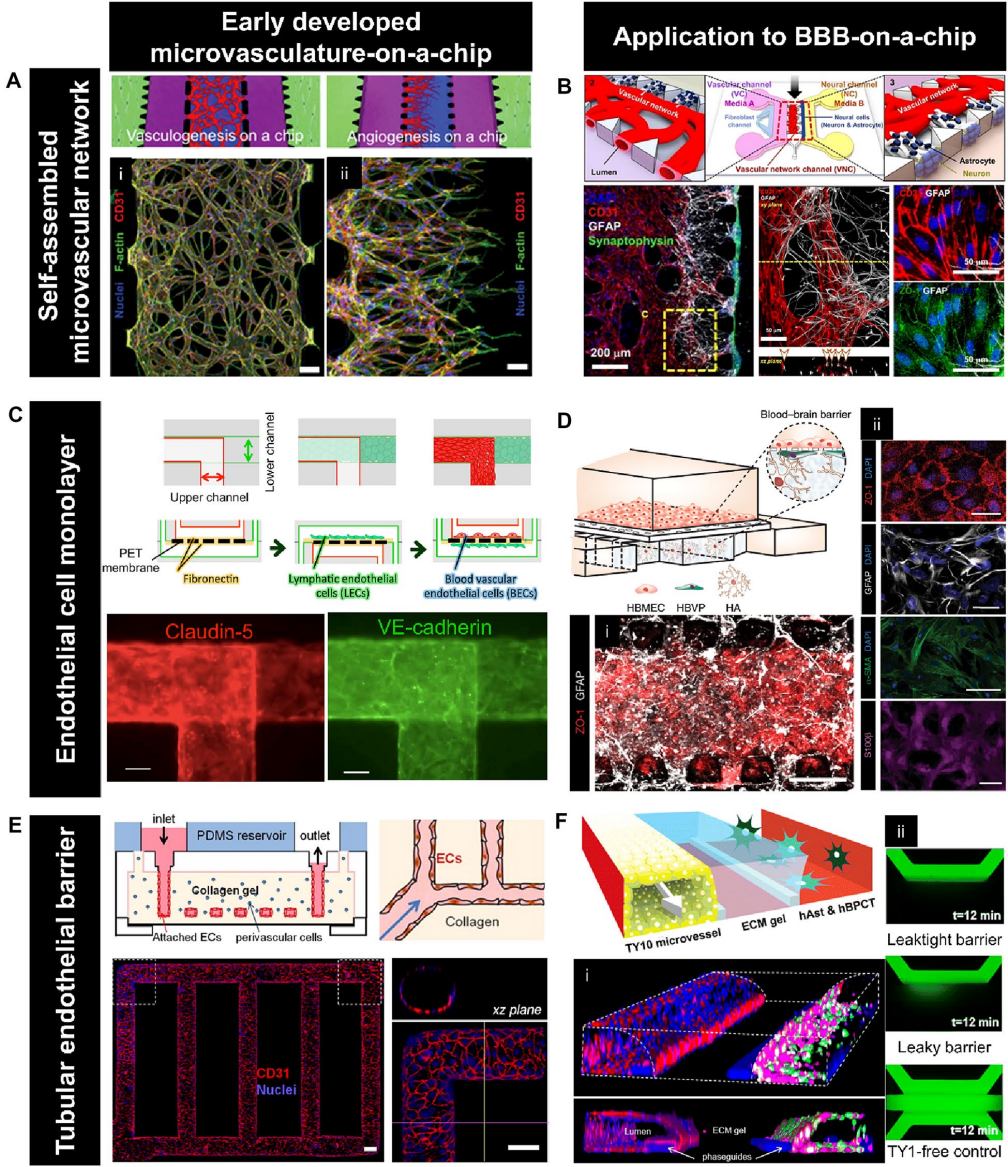
Microvasculature-on-a-chip technology. **a** Microfluidic platform to reconstruct a self-organized microvascular network. Configurations and confocal microscopy images presenting the microvasculature constructed by (i) vasculogenesis (scale bar: 100 μm) and (ii) angiogenesis (scale bar: 20 μm) at day 4. [62]. **b** Self-assembled BBB microvascular network. Schematic diagram of BBB-on-a-chip consisting of endothelial cells, astrocytes (ACs), and neurons (top). Confocal microscopy images of astrocytes (GFAP, white), neurons (Synaptophysin, green), and microvasculature (CD31, red) (bottom-left). Immunostaining of CD31 (red) and tight junctions, ZO-1 (green) of the vascular network—astrocyte interface (bottom-right). [63]. **c** Microfluidic device to culture endothelial barriers. Configurations of blood-lymphatic endothelial cells layer culture (top). Immunostaining of an endothelial-specific protein, claudin-5, and an endothelial-specific adhesion molecule, VE-cadherin (bottom). Scale bar, 100 μm. [64]. **d** BBB structure emulated by the microfluidic culture of endothelial cells monolayer. Schematic representation of the microfluidic BBB model (top). (i) Confocal image of the bottom of the chip with endothelium (ZO-1, red) and network of astrocytes (GFAP, white). Scale bar, 50 µm. (ii) Tight endothelial monolayer (ZO-1, red; DAPI, blue), pericytes cultured under the porous membrane (α-SMA, green; DAPI, blue), and astrocytes labeled with GFAP (GFAP, white) and S100β (S100β, magenta). Scale bar, 50 μm [65]. **e** Tubular endothelial barrier-on-a-chip. Schematic representation of the microfluidic system(top). Confocal microscopy images of the overall endothelialized ductal structure and the magnified section of network corner (red, CD31; blue, nuclei) (bottom). Scale bar, 100 μm [66]. **f** Ductal structure of BBB reproduced by microfluidic technology. Cross-section view of the center of the chip for a three-lane coculture system of endothelial cells, pericytes, and astrocytes (top). (i) Z-stack confocal image of 3D reconstructed BBB (Calcein-AM, green and magenta; PECAM-1, red; nuclei, blue). (ii) Images of FITC-dextran (20 kDa) perfused in the microchannels [67]

The gradient of soluble angiogenic factors generated by the microfluidic system induced sprouting angiogenesis via the self-organizing process of seeded ECs [69]. Using a strategy to promote vasculogenesis and angiogenesis via secreted agents from cellular components, 3D functional microvascular networks can be built from ECs in microfluidic devices. For instance, a perfusable microvasculature was constructed through both vasculogenesis and angiogenesis of ECs embedded in fibrin gels supported by factors released from fibroblasts in the microfluidic device compartmentalizing each cellular portion using gel-trapping micropost structure [62] (Fig. 1a). The microfluidic platform with microposts also enabled to measurement of the permeability of lumenized microvessels generated by angiogenesis [70]. With a similar gel-trapping approach, the vasculogenesis-like process was supported by bone marrow-derived human mesenchymal stem cells, which release proangiogenic factors [71]. Also, the microvascular construct was generated by injecting fibrin gel loaded with normal human fibroblasts and endothelial colony-forming cell-derived ECs in each environmental condition: interstitial flow or hypoxic conditions [72]. The lumen structure constructed by this module was perfused by connecting to microfluidic channels to present an engineered anastomosis system [73, 74]. This platform exhibited flexibility to culture microvessels in high-throughput methods [75] integrating with multiple components, including tumor and cardiac muscle tissues [5].

The self-morphogenic microvascular engineering method has been applied to the studies for the effects of various stimulations and constituents on the microvasculature. The angiogenic sprouting response to mechanical cues, including shear stress [76], interstitial flow [77, 78], magnetic stimulation [79], and compression [80], was investigated using the self-organized microvasculature models. In the microvascular environment, the angiogenic sprouting was induced when a shear stress threshold was surpassed [76]. The interstitial flow promoted angiogenic sprouting when the flow direction was opposite from sprouting, while the flow given in the same direction inhibited the sprouting in the microfluidic platform [77]. Also, low interstitial flow in the microfluidic system eliminated the spatial gradients of morphogen and maneuvered angiogenic growth [78]. Magnetic bead movement enhanced microvessels growth [79], while compressive force increased reactive oxygen species and vascular leakiness [80]. The response of the microvascular plexus subjected to other types of stimulation caused by nanoparticles [81–83], anti-neovasculogenic agents [84], fine particulate matter [85], and airborne nanoscale particles [86] was examined using the microfluidic models. Furthermore, the cellular component and ECM are also important cues to modulate the microvascular environment. For example, the mature and functional vascular network was established by the self-morphogenesis process of induced pluripotent stem cell (iPSC)-derived ECs in synthetic hydrogels [87] and the fibrin gel [88]. Also, this platform allowed to investigate the interaction between perivascular cells and microvascular constructs. Specifically, the microfluidic platforms demonstrated the contribution of pericytes to inhibiting the enlargement of microvessels and constructing a dense network [89] and the connecting mechanism between the vessel-like structures in the spheroid of human lung fibroblasts and the sprouts of microvessels [90].

Beyond engineering each component and stimulation, the self-organized microvasculature-on-a-chip techniques were applied to recapitulating microvascular systems in multiple organs. For instance, angiogenesis in bone was reconstituted by culturing angiogenic sprouts in a mineralized ECM consisting of hydroxyapatite [91] and inducing bone marrow angiogenic process by leukemia cells [92]. Besides, the subcutaneous blood vessels replicated by coculturing human ECs with dermal fibroblasts and keratinocytes exhibited enhanced angiogenesis during exposure to skin-irritation agents, which was proposed as an in vitro skin-irritation model [93]. The function of microvasculature in the brain, such as the brain-blood barrier (BBB) and blood-retinal barrier (BRB), was emulated using this type of microfluidic model [63, 94–98]. BBB-on-a-chip was developed by integrating various types of ECs with cellular constituents in the BBB microenvironment, such as astrocytes, pericytes, and neurons. The microfluidic coculture system consisting of astrocytes, neurons, and human umbilical vein endothelial cells (HUVECs) enabled to the reproduction of low permeability of the vasculature, which is one of the critical physiological features of BBB by adjusting media conditions [63] (Fig. 1b). Furthermore, BBB structures exhibiting physiological characteristics of BBB, including enhanced junction proteins, were engineered by combining astrocytes and pericytes with ECs such as human brain microvascular ECs [94] and iPSC-ECs [95]. Using this approach using iPSC-ECs, the transport of polymer nanoparticles across BBB was evaluated [96]. Moreover, the outer BRB mimicked in microfluidic chips shows the anatomical structure of outer BRB with higher trans-epithelial electronic resistance value than the value of the epithelial monolayer [97] and the pathophysiological process of age-related macular degeneration [98].

For high-throughput culturing and drug screening systems of self-assembled microvascular network in vitro models, facilitating 3D gel patterning to encapsulate endothelial and other cells is one of the most required manipulations. To allow for simple gel trapping, open microfluidics was employed to injection-molded microfluidic chips, which enables to mimic the BBB construct [99], tumor microvasculatures [100], and the ocular neovascularization [101].

### Endothelial cell monolayer

One of the structural characteristics of the blood vessel is that ECs are aligned in a single layer in contact with circulating blood. A monolayer of ECs, known as the endothelium, is one of the essential architectures in the human body system. This structure regulates multiple physiological processes such as immune mechanisms, the transport of blood cells, and the control of vascular tone [102]. The input from the microenvironment such as biomechanical forces, including shear stress and cyclic strain, determines the endothelial phenotype [103]. To reflect the features of the endothelium on in vitro models, the microfluidic system which provides the dynamic environment has been employed to reconstruct the EC monolayer. Controlling the microfluidic condition in each EC culture system enabled a long-term culture of the ECs layer, which maintains cellular viability and promotes the expression of the EC marker proteins as well as adhesion molecules elevated by the effects of the inflammatory cytokines [104].

Thanks to the strategy, a wide range of barrier functions of numerous organs such as the lung [105–108], lymphatic vascular system [64], placenta [109–113], skin [114], brain [65, 115, 116], and gut [117] has been emulated in vitro. A lung model is composed of an alveolar epithelium and an endothelium on the opposite sides of a porous membrane and is subjected to a respiration-mimetic dynamic motion controlled by a vacuum system [105]. This microfluidic device facilitated reproducing the effects of physiological breathing on nanoparticles uptake [105] and drug-toxicity-induced pulmonary edema progression, incorporating the development of vascular leakage [106]. A lung-on-a-chip array was also developed by exposing the endothelial and epithelial layer on both sides of the porous membrane to cyclic strain using an electro-pneumatic set-up [107]. Using the alveolar-capillary interface barrier built by patterning human pulmonary alveolar epithelial cells and ECs on the opposite sides of the Matrigel membrane in the microfluidic chip, the pulmonary response to air pollutant PM2.5 were assessed [108]. A microcirculation system consisting of blood and lymphatic vessels was reproduced by coculturing both blood and lymphatic ECs on the porous membrane under flow conditions, which promote endothelial cell-cell junctions [64] (Fig. 1c). Moreover, a multi-layered structure of human trophoblasts and ECs on a porous membrane subjected to dynamic flow mimics the architecture of the human placental barrier and physiological transport of glucose [109, 110] and drug [111] across the maternal-fetal interface. The placental inflammatory responses with bacterial infection were investigated via the reconstructed placental barrier using a similar approach [112]. Also, the placental transport of nanoparticles was examined using a placental barrier-on-a-chip fabricated by coculturing trophoblasts and ECs on the opposite sides of ECM [113]. The interlayer interaction provided by the microfluidic system allowed for the reconstitution of the complex skin structure consisting of epidermal, dermal, and endothelial layers, where inflammation and edema were induced in this model [114]. The neurovascular construct established by coculturing neurons, astrocytes, and ECs layers vertically patterned on gel walls under dynamic flow exhibited size-selective permeability [115]. Particularly, the function and morphology of BBB, including low permeability of the barrier and the expression of efflux pumps and transporters, were recapitulated by culturing ECs layers interfaced with astrocytes and pericytes under shear flow [65, 116]. The BBB structure engineered by brain-like microvascular ECs derived by iPSC technology demonstrated the improved barrier function under hypoxic conditions [116]. Besides, the barrier architecture consisting of human microvascular ECs allows for neuroinflammation modeling by establishing an astrocytic network with reduced reactive gliosis markers while enables nanoparticles transport testing [65] (Fig. 1d). The human gut-vessel microenvironment with a peristaltic motion was reconstituted by culturing intestinal epithelial cells-ECs under a pneumatic pumping system, which emulates the intestinal barrier damage and inflammatory reactions caused by *E. coli* [117].

Numerous microengineering technologies serve as a tool to construct microfluidic platforms to replicate endothelial barriers [118–120] and evaluate the barrier function [121, 122]. For instance, a semipermeable and optically transparent membrane created by a hydrogel engineering technology enabled to promote endothelial cellular adhesion and growth under microfluidic flow while compartmentalizing the microenvironments [118]. A pumpless microfluidic device designed for directional perfusion provided continuous fluid flow driven by gravity to organize extensive endothelial barriers [119]. Also, a hydrophobic-patterning technique facilitated gel loading in the microfluidic platform without microposts to guide the route, where a confluent EC monolayer was constituted on the patterned gel wall [120]. To assess the mechanical and chemical effects, including shear stress on the endothelial barrier culture, the microfluidic platforms were designed to measure the permeability of the ECs layer using tracer molecules with varying sizes [121] and transendothelial electrical resistance [122]. Furthermore, the influence of external substances such as chemoattractant [123] and gold nanoparticles [124] on the microenvironment of endothelium under fluid flow was investigated using engineered microfluidic platforms.

### Tubular endothelial barrier

The microcirculation operated by distributing blood to tissues through ductal structures of vessels plays a vital role in human pathophysiology. Notably, the mechanical and rheological properties determined by multiple variables such as blood flow, blood cell behaviors, and interaction between blood cells and the vascular wall during ducal flow considerably affect the microcirculatory system [125]. Thus, the tubular structure of microvasculature is crucial to understand the hemodynamics [126] and the dysfunction of the system [127], which is important for therapeutic developments. In vitro microfluidic models to reconstruct the tubular lumen structures of endothelial barriers have been established to implement the strategies to explore unaddressed questions related to the pathophysiology [128].

Multiple types of engineering techniques were employed to fabricate fit-for-purpose microfluidic platforms to build the ductal structure of the ECs layer. To reproduce the vascular disorders induced by pathological oxygen stress and shear flow, the ECs layers on the fibronectin-coated walls of tubular microchannel were cultured in hypoxic conditions under fluid flow [129]. Through a hydrogel casting process using a 3D printing technique, a confluent ductal layer of ECs was constructed on the microchannel walls consisting of gelatin methacrylate (GelMA) [130]. A microfluidic model to recapitulate angiogenesis, tubulogenesis, and anastomosis developed from tubular microvascular structure also utilized a photo-crosslinkable material, GelMA [131]. On the other hand, ductal microvasculatures-on-a-chip manufactured using photo-degradable hydrogels allowed for geometrical control [132]. A molding process using the microwire also enabled the controllable design of tubular structure to emulate vascular compliance and topography [133]. Moreover, the microfluidic tubular vasculature model, which can be applied to the anastomosis, was developed by integrating a synthetic biodegradable polymer with 3D stamping technology [134]. The in vivo-like ducts were also built by aligning smooth muscle cells and ECs on microwrinkled circular microchannels constructed via the soft-lithographic molding process [135].

To examine the behavior of blood cells and blood flow during the microcirculation, a certain type of blood cells such as red blood cells (RBCs) and white blood cells were inserted in the tubular endothelial barriers-on-a-chip. For example, RBCs were introduced to the perfusable capillary tube, which was created by fusing migrating ECs from opposite ends along patterned fibrin gels in the microchannels [136]. Besides, the effects of endothelium on the behavior of RBCs around the endothelial wall under hydrodynamic resistance were examined using ductal endothelial microchannels built by the soft-lithography technique [137]. RBCs are also perfused in ECs layer-covered microchannel under mechanical stress to mimic damage on the pulmonary microvasculature that occurred during RBCs transfusion and breathing [138]. Particularly, an agarose-gelatin interpenetrating polymer network hydrogel was utilized to fabricate a tubular endothelialized fluidic system, in which sickle RBCs were loaded in the channels to investigate the endothelial barrier dysfunction in sickle cell disease [139]. Moreover, neutrophil, one of the types of white blood cells, was introduced in the endothelial tubes constructed in microfluidic channels with varying bifurcation angles [140] and mold-casted microchannels [141, 142] to examine the interaction between endothelium and neutrophils. Beyond injecting each type of blood cells, whole human blood was infused into the tubular structure of endothelial barriers. Whole blood was inserted through the cylindrical ECs barriers on microchannels fabricated using a biocompatible sacrificial molding [143]. The collagen scaffolds to form tube-shaped microvessels were manufactured via a molding process and utilized to examine the prothrombotic state under the inflammatory stimulation by injecting human blood into the ducts [66] (Fig. 1e). Likewise, more adhered monocytes on the inner endothelial surface of the lumen were investigated in the presence of an inflammatory cytokine than in the absence of the stimulation using the endothelial ducts in microfluidic channels manufactured by 3D stamping technology [144]. Also, platelet aggregation was observed when whole blood was perfused in 3D printed microfluidic chips with a stenotic geometry [145] and constricted microchannels generated by the collagen-patterning method emulating stenosis in atherosclerosis [146]. Interestingly, a bleeding model was developed by coupling the microfluidic chip fabricated by soft lithography with a pneumatic valve to induce vascular injury of endothelial barriers, which allowed for the investigation of the effect of antiplatelet agents on clot retraction and hemostatic plug formation [147].

Recent studies have also presented the application of the tubular ECs layer-on-a-chip technology to replicating the microvascular system in other organs such as skin [148] and BBB [67]. For instance, a skin-equivalent model was established by constructing the ductal microchannel in the collagen scaffold containing dermal fibroblasts and epidermal keratinocytes, coating the inner surface of the channel with ECs and perfusing media using a pumping system [148]. Furthermore, tubular BBB structure with adherens and tight junctions was reproduced by coculturing ECs, astrocytes, and pericytes in the commercially available microfluidic platform, which was utilized to test antibody transcytosis [67] (Fig. 1f).

## Microfluidic approach to reconstitute vascularized solid tumors

### On-chip tumor spheroids formation using microfluidics

**Fig. 2:**
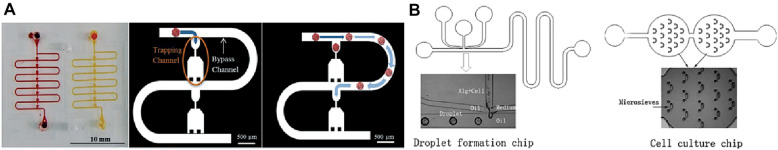
Microfluidic systems for tumor spheroid culturing. **a** A microfluidic device that utilizes hydrodynamics to guide cancer cells into U-shaped traps and form tumor spheroids. This method highlights the controllability over the number of cells in each spheroid, the ability to sustain long-term culturing, and on-chip drug testing, which are features not present in conventional methods [149]. **b** A tumor spheroid culturing device that combines microfluidic principles and cell suspension techniques. The cell suspension injected through the PDMS channel was gathered in the pyramid-like holes to be aggregated. The device supports spheroid formation and long-term culture [150]

A tumor spheroid is a 3D cell cluster with a spherical structure derived from a variety of tumor cell lines or even with other tumor-related cells [151]. Tumor spheroid formation became of interest because they better recapitulate the in vivo TME, and resultantly, provides a more accurate platform for biological studies and therapeutic testing. Tumor cells are formed into spheroids to better replicate the in vivo TME. This allows for more accurate biological studies and therapeutic testing. Conventional tumor spheroid forming techniques include hanging drop [152], liquid overlay [153], spinner flask [154], and NASA’s rotating wall vessel (RWV) [155]. In addition to formation, these techniques also accommodate drug testing and performance analysis. Since tumor spheroids, Nevertheless, the conventional methods have their drawbacks as the dynamic culturing systems (spinner flask and RWV) require large volumes of media, and the static culturing systems (hanging drop and liquid overlay) are laborious and require frequent media exchanges [151]. To overcome these challenges, microfluidics has become a popular solution that commonly includes hydrodynamics [149], hydraulic resistance [156], droplet [157], and microwells [150] for tumor spheroid culturing (Fig. 2). Microfluidics advanced spheroid culturing techniques by providing higher controllability over spheroid size and growth parameters, continuous perfusion, and faster formation [158].

### Applying 3D tumor spheroids to microvasculature-on-a-chip devices

Organs-on-a-chip approaches have extended their potentials to reconstructing TME [159–161]. Furthermore, to replicate the crucial features of the microvascularized tumor complex, such as tumor angiogenesis and metastasis [162–165], the microfluidic tumor-on-a-chip incorporating a microvascular compartment has been developed [166]. The self-assembly microvascular engineering approach has facilitated to reconstruct tumor angiogenesis [62, 167] and tumor vasculature for analysis of patient-derived tumor cellular behaviors on the network [168] and anti-cancer drug testing [100, 169–171]. This self-organization method has also been applied to emulate metastatic cascade such as extravasation of tumor cells [172–175], including when the TME is in the bone matrix [176] or hypoxic condition [177]. As another technique to reconstitute microvasculature, the endothelial monolayer organized with tumor cells in the microfluidic platform has provided a reliable module to recapitulate tumor behaviors on the endothelial barrier [178], including metastatic response [179, 180] such as transendothelial invasion [181, 182], intravasation [183–186], and extravasation [187]. Moreover, integrating tubular endothelial barriers-on-a-chip with cancer cells has enabled to the investigation of transvascular migration of tumor cells [188] and assess anti-drug efficacy [189] and nanoparticle extravasation [190] in the microfluidic model. As described above, considerable progress in translational research to understand vascular oncology has been achieved by combining single tumor cells with microvasculature-on-a-chip techniques.

Nevertheless, the aforementioned models present constraints in emulating the pathophysiology of solid tumor tissue with microvascular plexus. To understand the microenvironment of solid tumor microvasculature is crucial because solid tumor with the abnormal microvasculature leads to regions of hypoxia and acidity [191, 192]. These unique physiological characteristics influence drug resistance to chemotherapy and anti-angiogenic factors, which is critical in cancer treatment [193–196]. 3D tumor tissues assembled in a microsphere, known as tumor spheroids, have been orchestrated with microvasculature-on-a-chip to address the challenges. To date, the microfluidic platforms to culture engineered tumor spheroids with the microvascular system have been developed (Table. 1) to reconstitute microvascularized solid tumors of numerous organ types, including lung, colon, brain, ovary, stomach, and breast. Unlike the microfluidic models depicted in Table. 1, where 3D tumor spheroids cultured in separate platforms were incorporated in microvasculature-on-a-chip, the technologies to assemble solid tumor tissue and construct a microvascular structure in the microfluidic platform at once have been developed [197–200]. The innovative combination of solid tumor tissue formation techniques and microvasculature-on-a-chip technologies has contributed to translational cancer research.

**Table 1:** Summary of 3D tumor spheroid—microvascular-on-a-chip models

Tissue	Cell sources (EC/Spheroid)	Approaches (Vascularization/Spheroid formation)	Features	Refs
Lung	HUVECs/A549	Endothelial cell monolayer/Ultra-low attachment dish	Investigated dispersion of cancer cells and increased EMT marker in the model, tested drug targeting EMT and analyzed cellular response	[201]
Colon	ECFCs/SW620 with ECFCs	Self-assembled network/Non-adherent round-bottom 96-well plate	Constructed vascularized breast and lung spheroids with robust sprouting and a lumenized vessel network and analyzed increased tumor cell intravasation as oxygen decreased	[202]
Brain	HDMECs/U87MG	Self-assembled network/Hanging drop-cell culture dish	Reconstituted the capillary beds-tumor tissue interaction using an “open-top” microfluidic chip and compared the vascular architecture with spheroid with the one without spheroid	[203]
Colon	HUVECs and HDLECs/SW620 with NHLFs	Self-assembled network/ Hanging drop	Reconstructed simultaneous angiogenesis/lymphangiogenesis to tumor-stroma spheroid and observed the physical interactions between the sprouts and tumor cells	[204]
Ovary	HUVECs/SKOV3	Endothelial cell monolayer/ 96-well plate precoated with agarose	Investigated no significant cellular uptake differences of the targeted and untargeted nanoparticles in the microfluidic and in vivo model in contrast to the control condition	[205]
Colon and stomach	HUVECs/SW620 and MKN74 with NHLFs, each	Self-assembled network/ Microwell plate	Analyzed angiogenic sprouts in the mineralized colorectal/gastric TME by varying the concentration of hydroxyapatite	[206]
Brain	HUVECs/U87MG and HepG2	Self-assembled network/ Non-adherent round-bottom 96-well plate	Quantified the vascular network area, number of sprouts, and sprouting length after anti-cancer drug screening in tumor spheroid-induced angiogenesis	[207]
Lung	HUVECs/A549	Self-assembled network/ Non-adherent round-bottom 96-well plate	Investigated vascular responses to chemotherapy, including vascular architecture, endothelial apoptosis, and oxidative stress, as well as demonstrated the cytotoxic effect localized at the outer surface of the traditional spheroid model in contrast to the microfluidic model	[208]
Breast	HUVECs/MCF7	Tubular endothelial barrier/ Hanging drop-cell culture dish lid	Presented fast penetration of natural killer cells to the tumor spheroid compared with antibodies and enhanced cytotoxicity for the spheroid periphery using the combination therapy	[209]
Breast	HUVECs/MCF7 with NHLFs and HUVECs	Self-assembled network/ Non-adherent round-bottom 96-well plate	Demonstrated the adaptability of the model to other cancer cells (HepG2, MDA-MB-231, and SW620) and perfusability of the network, and analyzed the dose-dependent effects of chemotherapy on the response of the tumor spheroids using the model	[210]
Ovary and lung	HUVECs/SKOV3 and A549	Self-assembled network/ Non-adherent round-bottom 96-well plate	Constructed a 3D tumor spheroid-perfusable microvascular network interaction to examine the effects of chemotherapy, drug resistance, and molecular diffusivity in the microenvironment	[211]

HUVECs, human umbilical vein cells; EMT, epithelial-mesenchymal transition; ECFCs, endothelial colony-forming cells; HDMEC, human dermal microvascular endothelial cells; HDLEC, human dermal lymphatic endothelial cells; NHLFs, normal human lung fibroblasts

## On-chip approaches for microvascularized tumor organoids

As previously described, exploring the physiological interaction of microvessels and solid tumor tissue is considerably significant in understanding oncology and cancer therapeutics. Though the integration of 3D tumor spheroids with the microvascular system in vitro contributed to this understanding, the current unmet goals of the models are to recapitulate the highly complex TME and heterogeneity of each patient tumor. Beyond the in vitro system to encompass the cellular aggregates and the microvessels, multiplexed microfluidic systems have been developed by combining organoids technologies with microvasculatures-on-a-chip systems. The microvascularized 3D tissues retain their functions and features representing the original in vivo tissues and increase the complexity of the microenvironments incorporating the microvascular system.

**Fig. 3:**
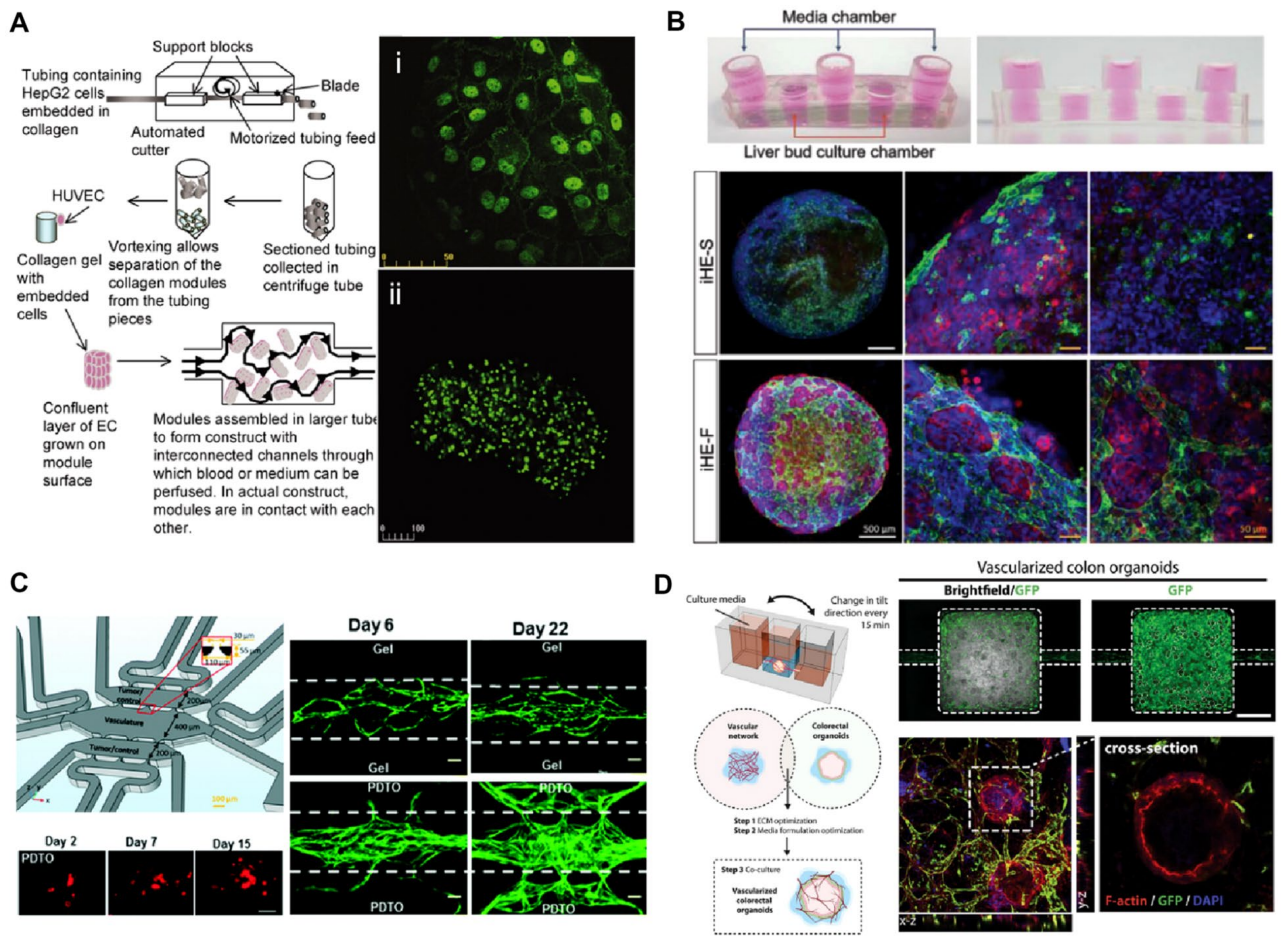
Modeling for vascularized organoids-on-a-chip. **a** The approach for collagen-HepG2 modules to build vascularized organoids. The HepG2 cells collagen cylinders covered with human umbilical vein endothelial cells (HUVECs) were cultured in the flow circuit enabling perfusion with medium or blood to deliver nutrients to the assembly. Confocal microscopy image of (i) vascular endothelial (VE)-cadherin staining of HUVEC layer on the construct and (ii) prelabeled viable HepG2 cells [212]. **b** 3D vascularized hepatic organoids in a rocker-actuated microfluidic system. Confocal images for CD31 (green) and albumin (ALB; red) of the liver organoids consisting of induced hepatic cells, HUVECs, and a decellularized liver extracellular matrix cultured under each condition demonstrate increased albumin expression and vascular network of the liver organoids when cultured with media flow: under static conditions (iHE-S) and dynamic conditions induced by the microfluidic system (iHE-F). Scale bars, 500 μm (white), 50 μm (yellow) [213]**. c** A microfluidic device to coculture patient-derived tumor organoids (PDTO) and a perfusable microvascular networks. Fluorescently tagged PDTOs (red; scale bar, 50 μm) cultured in the microfluidic chip grow in the pre-vascularized system. The vascular network (green; scale bar, 100 μm) cocultured with PDTOs show highly angiogenic features [214]. **d** A microfluidic platform to engineer vascularized colon organoids. Confocal microscopy images of vascularized colon organoids for F‐actin (red), DAPI (blue), and GFP‐endothelial cells (green). [215]

The microfluidic platform to engineer microvascularized organoids was demonstrated by providing the controllable fluid perfusion on the collagen cylinders consisting of liver cancer cells and surrounded by HUVECs [212] (Fig. 3a). Likewise, vascularized kidney organoids were constituted using pretubular aggregates differentiated from human pluripotent stem cell-derived metanephric mesenchyme cells under controllable flow on milifluidic chips [216]. The dynamic microenvironment in the platform contributed to enhanced endothelial maturity and vascularization with perfusable lumens as well as more mature podocyte and tubular portions. For a vascularized liver organoids modeling, induced hepatic cells were cocultured with HUVECs in a decellularized liver extracellular matrix loaded in a microfluidic platform with a rocker system [213] (Fig. 3b). Under the gravity-driven flow in the system, the liver organoids presented induced vascularization by HUVECs and extended albumin expression. In addition to the studies integrating normal organoids with vascular networks, patient-derived tumor organoids were loaded in the self-organized microvasculature-on-a-chip, although the tumor organoids lack the characteristics of 3D solid tumor tissue [214] (Fig. 3c). The microvascular network cultured with patient-derived tumor organoids presented highly angiogenic features. Using a customizable microfluidic platform, ECM components and the culture media composition were adjusted to coculture patient-derived colon organoids and a self-assembled microvasculature under intravascular perfusion [215]. This device was applied to the reconstitution of a colorectal inflammation with an innate immune function in the circulatory system was reconstituted in this device by applying monocytes and inflammatory cytokines to the microenvironment.

**Fig. 4:**
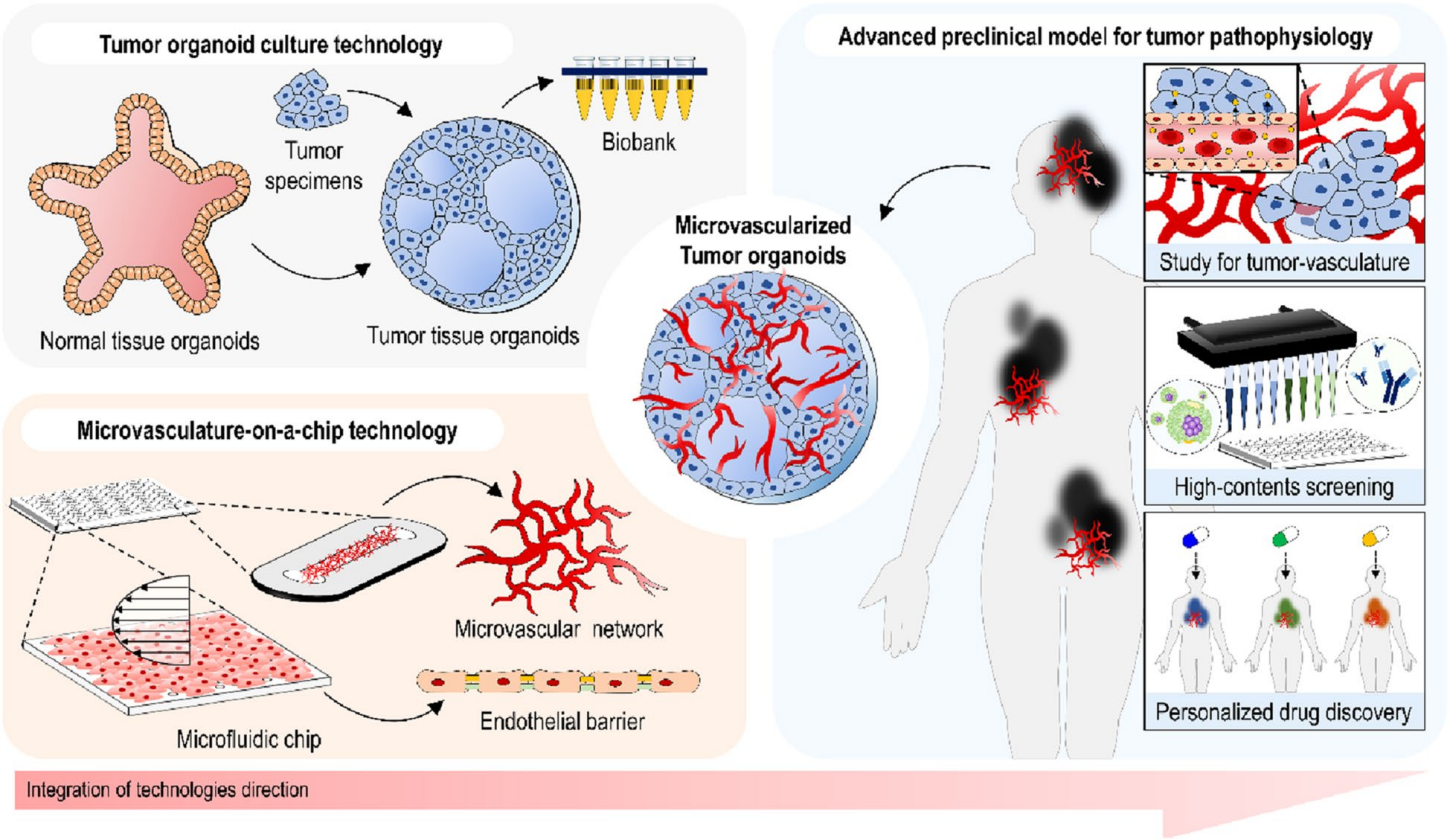
Microvascularized tumor organoids-on-a-chips principles

Upon embedment of functional organoids into microvasculature-on-a-chip, multiplexed microvascularized “on-a-chip” models will be constructed by addressing the key hurdles. Representation of an in vivo-like microenvironment using the platforms allows for the development of therapeutics and biological studies. Likewise, emulating vascularized TME by incorporating tumor organoids into microvascular beds contributes to the advance in cancer treatment and research (Fig. 4).

## Conclusions

The current organoid and organ-on-a-chip technologies have contributed to the recapitulation of human cancer pathophysiology. Although the tumor organoids and TME-on-a-chip techniques have provided promising tools for preclinical studies, multiple challenges such as mimicking the systemic delivery of anti-cancer drugs remain for advanced applications. One of the recent strategies is the microvascularization of tumor organoids, which allows to mimic a physiologically relevant molecular transport system to provide nutrients to tumor organoids or to deliver anti-cancer drugs, highlighting the importance of this technology integration.

However, several challenges still have to be overcome in microvascularized tumor organoids in terms of materials and design. First, media formulations and ECM components should be adjusted to induce microvascularization in tumor organoids during their organogenesis. Moreover, the development of alternative materials is required because polydimethylsiloxane, which is the most widely used material in the manufacturing of microfluidic platforms, severely adsorbs biomolecules to decrease the accuracy of drug testing. A new module for a highly controllable dynamic environment needs to be established to precisely control the organogenesis and microvascular growth. Following this, organ-on-a-chips with microfluidic systems could provide more precise control over the organogenesis than conventional methods. This microsystem can also directly lead to drug testing without further transfer or disturbance of the spheroids with minimal waste by easily integrating biosensors.

Here, we highlighted tumor organoids cultures, microvasculature engineering, and microfluidic techniques, which may serve as versatile tools to reproduce TME with pathophysiological relevance. The established perfusable tumor culture system incorporating microvascular networks with high functional resemblance to the patient tumor may allow for continuous growth and maturation of tumor tissue via microcirculation. This synergistic strategy to reconstruct microvascularized tumors may play a critical role in the development of personalized cancer therapy. Consequently, we expect that further studies to integrate the microvascularized tumor model with a complex mechanism, such as an immune system, enable the close reproduction of cancer pathophysiology.

## Data Availability

Not applicable.

## Abbreviations

TME: Tumor microenvironment
ECM: Extracellular matrix
PDTX: Patient-derived tumor xenografting
EC: Endothelial cell
HUVECs: Human umbilical vein endothelial cells
iPSC: Induced pluripotent stem cell
BBB: Brain-blood barrier
BRB: Blood-retinal barrier
GelMA: Gelatin methacrylate
RBC: Red blood cell
RWV: Rotating wall vessel
AC: Astrocyte
PC: Pericytes
HUVECs: Human umbilical vein endothelial cells
FSS: Fluid shear stress
iHE-S: Under static conditions induced by the microfluidic system
iHE-F: Dynamic conditions induced by the microfluidic system
PDTO: Patient-derived tumor organoids
EMT: Epithelial-mesenchymal transition
ECFC: Endothelial colony-forming cell
HDMEC: Human dermal microvascular endothelial cells
HDLEC: Human dermal lymphatic endothelial cells
NHLF: Normal human lung fibroblast
ALB: Albumin
PD-1: Programmed cell death protein 1
PD-L1: Programmed death-ligand 1
